# Synthesis, *In-Vitro* Activity and Metabolic Properties of Quinocetone and Structurally Similar Compounds

**Published:** 2017

**Authors:** Keyu Zhang, Chunmei Wang, Xiaoyang Wang, Haihong Zheng, Juan Zhao, Mi Wang, Sui Xiao, Chenzhong Fei, Wenli Zheng, Lifang Zhang, Feiqun Xue

**Affiliations:** *Key Laboratory of Veterinary Drug Safety Evaluation and Residues Research, Shanghai Veterinary Research Institute, Chinese Academy of Agricultural Sciences, Shanghai 200241, China.*

**Keywords:** Quinocetone, 2-Propenyl moiety, Cytotoxicity mechanism, Toxiccophore

## Abstract

To investigate the cytotoxicity mechanism of quinocetone from the perspective of chemical structure*,* quinocetone and other new quinoxaline-1, 4-dioxide derivatives were synthesized, and evaluated for their activities, and analysed for the metabolic characteristics. Quinocetone and other new quinoxaline-1,4-dioxide derivatives were synthesized, and evaluated for their activities, and analysed for the metabolic characteristics. The synthetic route started from 2-nitroaniline which was reacted with 3-bromopropanoic acid followed by the reaction of acetylacetone to afford 2-acetyl-3-methylquinoxaline-1, 4-dioxide. The aldol condensation of the later compound with aromatic aldehydes led to the formation of the quinocetone structure similar compounds. A number of prepared derivatives exerted antimicrobial activities and cytotoxicity potency. Analysis of metabolic pathways in vitro displayed 2-propenyl and N→O groups were the major sites. The results suggested 2-propenyl group exert important role in cytotoxicity of quinocetone and is another major toxiccophore for quinocetone, and different electronic substituents in arylidene aryl ring could affect the electronic arrangement of 2-propenyl and N→O groups to chang the cytostatic potency.

## Introduction

**Figure 1:**
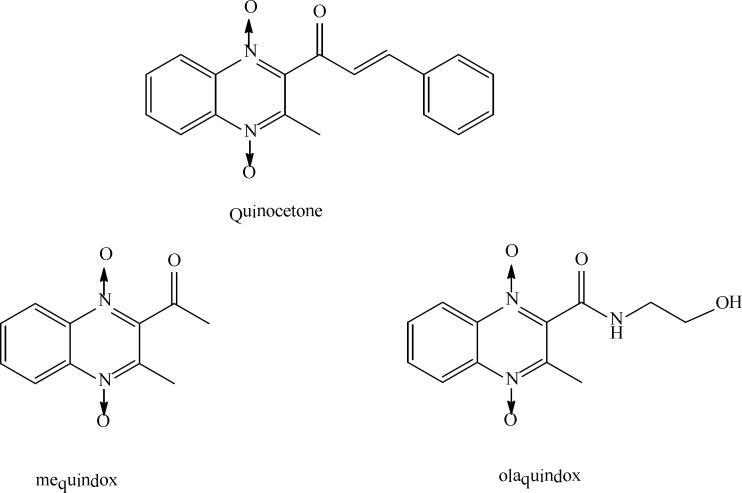
Structures of the quinoxaline-1,4-dioxides derivatives.

Quinoxaline is an organic heterocyclic compound that has been used as base of synthesis of bioactive derivatives and several investigation groups have demonstrated their potential in medical and pharmacological applications (1-4). These studies revealed that these compounds have potential anti-tumor, anti-bacterial, anti-fungal, and anti-viral applications (1). Quinocetone or 3-Methyl-2-(3-phenyl-2-propenoyl)-quinoxaline-1, 4-dioxide (CAS No. 81810-66-4,C_18_H_l4_N_2_O_3_) is a new synthetic member of quinoxaline-1, 4-dioxide derivatives (Figure 1) and has been approved as an animal growth promoter in China since 2003 (5). Other quinoxaline-1, 4-dioxide derivatives such as carbadox, olaquindox and mequindox (Figure 1), were widely used as antimicrobial growth promoting agents to improve production in food-producing animals (4, 6).

Based on the available data from the literature, quinoxaline-1,4-dioxides exert diverse toxicities, including mutagenicity, phototoxic, developmental, and reproductive toxicity and carcinogenicity in various test systems (4, 6-10). Now, more and more studies have indicated that the toxicity of quinoxaline-1, 4-dioxide derivatives are dependent on the presence of their N-oxide groups (11-13). Quinocetone should have similar cytotoxic as aloquindox, if the N-oxide groups are the unique factor that determines the toxicity of the compound, owing to quinocetone has the same parent nucleus structure as aloquindox. However, quinocetone has significant growth inhibitory effects on HepG2, Vero, L-02 and Chang liver cell lines *in-vitro* test systems, and the growth inhibition ratios of olaquindox is much lower than quinocetone (7,11). Therefore, we considered that characteristics of biological activity of quinocetone may be rooted in its special chemical structure and metabolic characteristics and proposed the hypothesis that 2-propenoyl in quinocetone is another major toxiccophore. 

Furthermore, concerning the safety of quinocetone in animal feeds increased (8, 11), investigation of the cytotoxicity mechanism of quinocetone will help assessing the risk of quinocetone in animal products. The investigation based on chemical structure, activity and metabolic characteristics may conduce to explain the cytotoxicity mechanism of quinocetone. In order to explore the hypothesis that 2-propenoyl is the major toxiccophore after N-oxide groups in the chemical structure of quinocetone, in this work, we have aimed to make a series of 3-methyl-2-quinoxalinbenzenevinylketo-1, 4-dioxides including quinocetone structure similar compounds and their deoxygenation by using Beirut reaction and aldol reaction, and investigated the biological activity and metabolic characteristics of these derivatives* in vitro*.

## Experimental

*General procedure for the synthesis*

The melting point (mp) of each compound was determined on a Stuart Scientific SMP3 melting point apparatus and was uncorrected. 1H (400 MHz) NMR spectra were recorded on a Bruker ARX 400 FT NMR instrument (400 MHz), The letters s, br s, d, t, and q refer to singlet, broad singlet, doublet, triplet, and quartet, respectively. IR spectra were recorded on a Thermo Fisher Scientific Nicolet-550 spectrometer, using KBr discs. Mass spectra were recorded on a Waters ACQUITY SQD Mass spectrometer. The progress of the reactions was monitored by TLC and visualized under UV illumination.

*2-acetyl-3-methylquinoxaline-1,4-dioxide 2*

The corresponding benzofurazan-1-oxide 1 (0.05 mol) was dissolved in acetylacetone (0.12 mol) at room temperature. And then triethylamine (22 mL) was added dropwise. The reaction mixture was stirred at room temperature for 24 h. The reaction was detected TLC. The resultant precipitate was collected by filtration, dried and recrystallized from ethanol to give 2. 

*2-(3-aryl-2-propenyl)-3-methylquinoxaline-1,4-dioxides 3a–h*

A mixture of 2-acetyl-3-methylquinoxaline-1, 4-dioxide 2 (0.009 mol) and an aryl aldehyde (0.015 mol) in methanol (15 mL) was stirred at 45~50˚C for 15~20 min. And then diethylamine (3 mL) was added dropwise. The reaction mixture was stirred at 45~50˚C for 4 h. The product formation was checked out using TLC. The solid which deposited was collected, washed with water and recrystallized from ethanol to provide the compounds in series 3. 

*2-acetyl-3-methylquinoxaline 4*

The corresponding 2-acetyl-3-methylquinoxaline-1, 4-dioxide 2 (0.09 mol) was dissolved in 1,4-diethylene dioxide (80 ml) and water (40 mL). And then sodium dithionite (0.4 mol) was added portionwise and stirred at 45~50˚C for 4 h. The product formation was checked out using TLC. The brown mixture was added to a lot of cold water to obtain solid. The resultant precipitate was collected by filtration, dried and recrystallized from ethanol to give 4. Data for 4. 

*2-(3-aryl-2-propenyl)-3-methylquinoxaline 5a–h*

A mixture of 2-acetyl-3-methylquinoxaline 4 (0.01 mol) and an aryl aldehyde (0.015 mol) in methanol solution (15 mL) was stirred at 45~50 ˚C for 15~20 min. And then diethylamine (3 mL) was added dropwise. The reaction mixture was stirred at 45~50˚C for 4 h. The product formation was checked out using TLC. The solid which deposited was collected, washed with water and recrystallized from ethanol to provide the compounds in series 5. 

*3-methyl-2-(3-fura-2-propenoyl)-quinoxaline-1,4-dioxide 6*

A mixture of 2-acetyl-3-methylquinoxaline-1,4-dioxide 2 (0.01 mol) and furaldehyde (0.015 mol) in methanol solution (15 mL) was stirred at 45~50˚C for 15~20 min. And then diethylamine (3 mL) was added dropwise. The reaction mixture was stirred at 45~50˚Cfor 4 h. The product formation was checked out using TLC. The solid which deposited was collected, washed with water and recrystallized from ethanol to give 6. 

*3-methyl-2-(3-fura-2-propenoyl)-quinoxaline 7*

A mixture of 2-acetyl-3-methylquinoxaline 4 (0.009 mol) and furaldehyde (0.015 mol) in methanol solution (15 mL) was stirred at 45~50 ˚C for 15–20 min. And then diethylamine (3 mL) was added dropwise. The reaction mixture was stirred at 45~50˚C for 4 h. The product formation was checked out using TLC. The solid which deposited was collected, washed with water and recrystallized from ethanol to give 7. 

*3-methyl-2-quinoxaline-2-carboxylate-1,4-dioxide 8*

The corresponding benzofurazan-1-oxide 1 (0.04 mol) was dissolved in ethyl acetylacetate (0.05 mol) at 0~5˚C and then morpholine (15 mL) was added dropwise. The reaction mixture was stirred at 0~5˚C for 4 h. The product formation was checked out using TLC. The resultant precipitate was collected by filtration, dried and recrystallized from ethanol to give 8. 

*3-methyl-2-quinoxaline-2-carboxylate 9*

The corresponding 3-methyl-2-quinoxaline-2-carboxylate-1, 4-dioxide 8 (0.007 mol) was dissolved in water (10 mL). And then sodium dithionite (0.025 mol) was added portionwise and stirred at 45~50˚C for 4 h. The product formation was checked out using TLC. The brown mixture was extracted with ethyl acetate (3 × 20 mL). The ethyl acetate extract was washed with brine and dried over anhydrous magnesium sulfate. The product was obtained by evaporation of the solvent and recrystallized from ethanol to give 9. 

*3-methyl-quinoxaline-2-carboxylic acid 10*

A mixture of 3-methyl-2-quinoxaline-2-carboxylate 9 (0.01 mol) and sodium hydroxide solution (0.5 mol/L, 20 mL) was stirred at 55˚Cfor 30 min. The product formation was checked out using TLC. Dilute hydrochloric acid (10% w/v) was added dropwise to adjust pH to 5 ~ 6, then standing 30 min. The pink solid which deposited was collected and recrystallized from ethanol to give 10. 

*3- methyl-quinoxaline-1,4-dioxide 11*

The corresponding benzofurazan-1-oxide 1 (0.033 mol) was dissolved in morpholine (10 mL) at room temperature. And then acetone (20 mL) was added dropwise. The reaction mixture was stirred at 50~55˚C for 4 h. The product formation was checked out using TLC. The resultant precipitate was collected by filtration, dried and recrystallized from ethanol to give 11. 

*Solvent and chemicals*

Olaquindox (n-(2-hydroxyethyl)-3-methyl-2-quinoxalinecarboxamide 1,4-dioxide, 99.78%) was obtained from the China Institute of Veterinary Drug Control (Beijing, China). DMEM culture medium and Fetal Bovine Serum (FBS) were obtained from Life Technologies (Carlsbad, California, USA). Acetonitrile (LC grade) was obtained from Fisher Chemicals (Fairl Lawn, NJ, USA). Ethyl acetate and other analytical reagents were of analytical grade and purchased from Shanghai experimental reagent co., LTD (Shanghai, China). Ultra-pure water was conducted by a Milli-Q water purification system (Millipore, Milford, MA, USA).

All test compounds were dissolved in dimethyl sulfoxide (DMSO, Sigma, USA) and then diluted in Dulbecco’s modified Eagle’s medium (DMEM, Gibco, USA) at desired concentrations for cell proliferation analyses or diluted in nutrient broth medium for antimicrobial susceptibility testing respectively. The final concentration of DMSO in the DMEM culture medium was under 0.1% (v:v).

*In-vitro antibacterial activity analyses*

The preliminary antibacterial activities were obtained for selected compounds against various bacteria, namely: *Staphyloccocus aureus* ATCC25923 (Gram-positive bacteria), *Clostridium perfringen *CVCC52,8604 (anaerobes bacteria),* Salmonella pullorum* (Gram-negative bacteria), *Escherichia coli * ATCC25922 (Gram negative bacteria) and *Aeromonas hydrophila* (Gram-negative bacteria) were used in the study. Each strain was cultured in appropriate broth medium. *Clostridium perfringen* was incubated in an anaerobic chamber. The minimum inhibitory concentration (MIC) for each test compound was estimated using the broth dilution method. The MIC’s was determined for each strain/compound pairs that presented antimicrobial activity. The method was modified from Mohapatra›s methods (14, 15). Cell suspensions were prepared in 2 mL of liquid medium with concentrations of various tests of compounds in 15 mL culture tubes by inoculation with 100 μL of 10^6 ^cfu/mL from each stock. The cultures were incubated at 28 ˚C or 37˚C for 24 h. Tubes showing no visible turbidity represented the MIC, and subsequently, were inoculated on the right side of the 9cm sterile nutrient agar plates without various test compounds and incubated for 24 h (Clostridium perfringen was incubated in an anaerobic chamber.). The least concentration, at which no growth was observed, represented the minimum bactericidal concentration (MBC) (16). 

*Cell culture*

HepG2 and normal human Chang liver cells were obtained from the Institute of Biochemistry and Cell Biology, Shanghai Institutes for Biological Sciences, Chinese Academy of Sciences (Shanghai, China). HepG2 and normal human Chang liver cells were cultured at 37˚Cwith 5% CO_2_ in DMEM supplemented with 2% L-glutamine, 10% fetal bovine serum (Gibco, USA), 100 U/mL penicillin and 100 U/mL streptomycin (Gibco, USA). 

*In-itro Cytotoxicity analyses*

The methodology for evaluating the cytotoxicity inhibitory activity of various test compounds towards cell proliferation has been described previously (11, 17). Cell proliferation inhibitory analyses were carried out using the 3-(4,5-dimethylthiazole-2-yl)-2,5-diphenyl tetrazolium bromide (MTT, Sigma, USA) assay. Briefly, cancers HepG2 and normal human Chang liver cells were harvested and resuspended in fresh growth medium. A 100 μL cell suspension (5 × 10^4^ cells/mL) was aliquoted into each well of a 96-well culture plate. After 12 h incubation, the medium was replaced with fresh medium containing various concentrations of test compounds. The cells were then incubated for another 24 h. At the end of exposure, 10 μL of MTT solution (5 mg/mL) was added, and the cells were incubated for 4 h at 37˚C. The cells were treated with 100 μL DMSO, and absorbance was recorded at 570 nm using a microplate ELISA reader (ELX800, Bio-Tek, USA). All experiments were performed at least thrice, and data were presented as the mean of sixplicate wells ± standard deviation (SD). Cell inhibition was calculated as follows: 

(OD of control group − OD of experimental group) / (OD of control group − OD of blank group) ×100%. The IC50 value for a compound was calculated using SPSS.

*Identification of metabolic pathways in HepG2 cells*

Preparation of sample with HepG2 cells

For recognition of the metabolic characteristics of quinocetone (3a), deoxyquinocetone (5a), olaquindox, 3-methyl-2-(3-fura-2-propenoyl)-quinoxaline -1,4-dioxide 6, and 3-methyl -2-quinoxaline-2-carboxylate-1,4-dioxide 8, HepG2 cells were seeded at 6× 10^4^ cells/ml density in 24-well plates. After 24 h incubation, the medium was replaced with fresh medium containing various concentrations (20~80 µN) of test compounds. After 24 h incubation, the medium was collected and extracted with ethyl acetate then centrifuged at 14000 g/min for 20min at 4˚C. The organic supernatant was evaporated under nitrogen atmosphere at 40˚C, the residue was dissolved in 200μL acetonitrile. The aliquot was filtered by a 0.22 μM membrane then analyzed. The blank is conducted in the absence of pharmaceuticals.

*Instruments and conditions of metabolic analysis*

All experiments were performed with a Waters Alliance 2695 LC system (Waters) and a quattro micro API quadruple mass spectrometer (Micromass, Manchester, UK) equipped with an electro-spray ion source (ESI). 

LC analysis was carried out on an XTerra C_18_ HPLC column (2.1 mm×100 mm, 3.5 μM) (Waters, Milford, MA, USA) equipped with a guard column (XTerra C_18_ 2.1 mm×100 mm, 3.5μM) (Waters, Milford, MA, USA). The mobile phase consisted of A (water containing 0.1% formic acid, v/v) and B (acetonitrile) with a gradient elution: 0–2 min, 95% A; 2–10 min, 95%-50% A; 10.01 min, 95% A. The flow rate during the complete runs was 0.2 mL/min. The injection of the pretreated sample was 10 μL and the column oven temperature was set at 30˚C.

**Figure 2:**
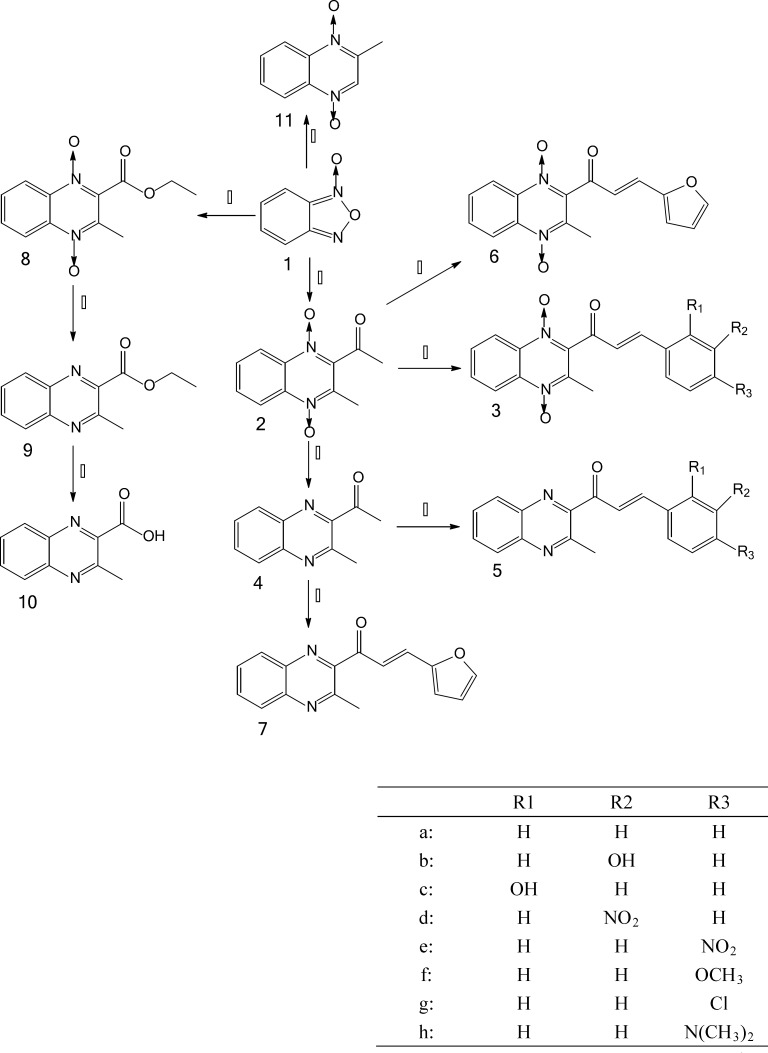
The reagents used in the syntheses of compounds are as follows, namely (ⅰ) acetylacetone/ triethylamine, (ⅱ) aryl aldehyde/ diethylamine, (ⅲ) sodium dithionite, (ⅳ) furaldehyde/ diethylamine, (ⅴ) ethyl acetylacetate/ morpholine, (ⅵ) sodium hydroxide, (ⅶ) acetone/ morpholine

**Figure 3:**
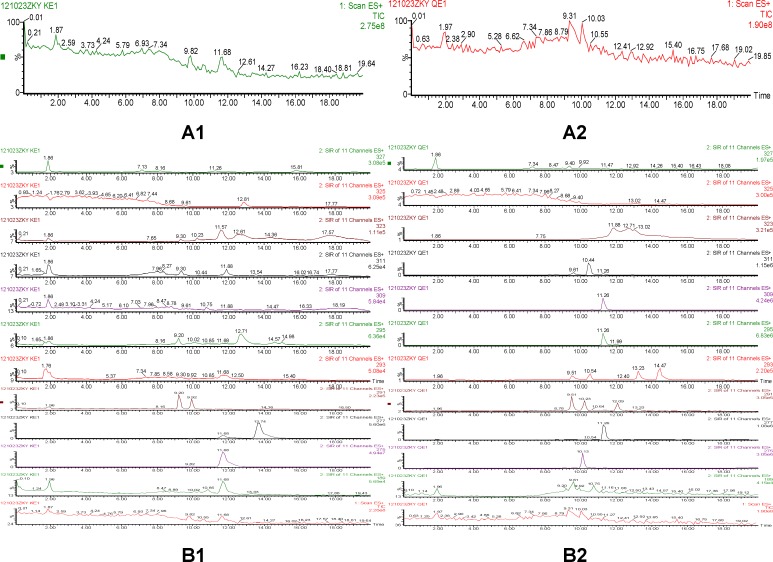
Total ion chromatogram and selected ion recording of quinocetone 3a in HepG2 cells incubated for 24h (A: Total ion chromatogram, A1 for the control group, A2 for the drug group; B: Selected ion reaction spectras, B1 for the control group, B2 for the drug group

**Figure 4:**
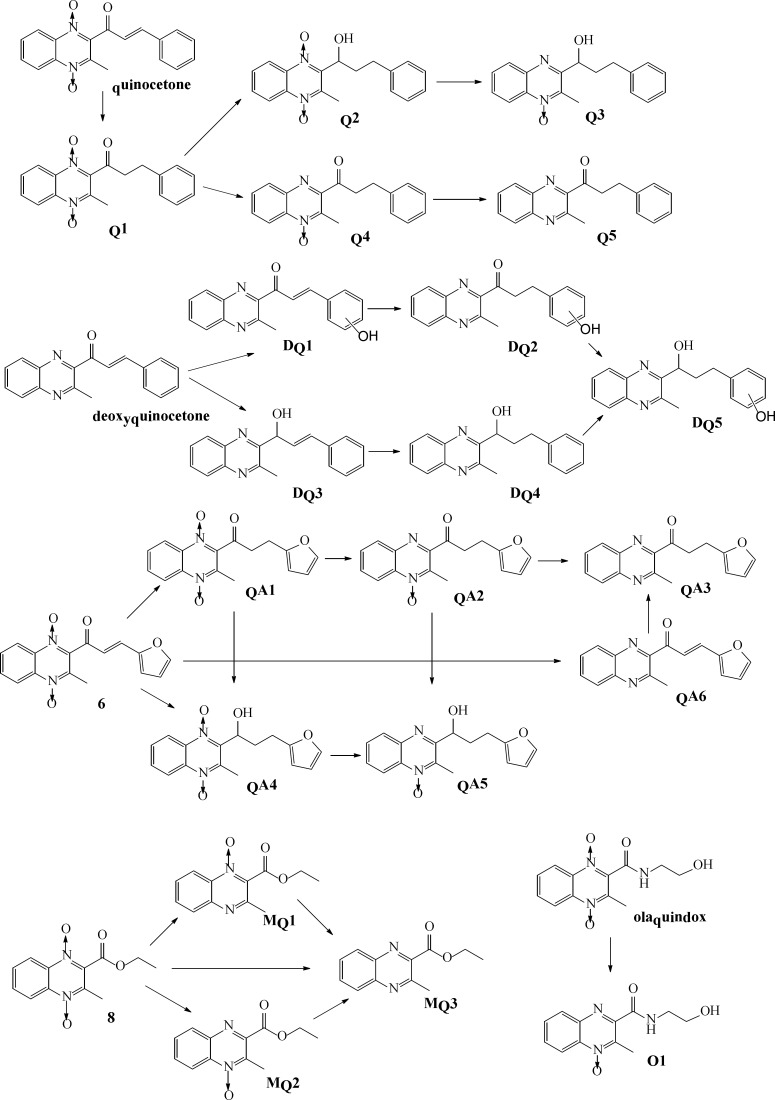
The proposed metabolic pathways of quinocetone 3a, deoxyquinocetone 5a, olaquindox, compound 6 and 8 in HepG2 cells

**Figure 5:**
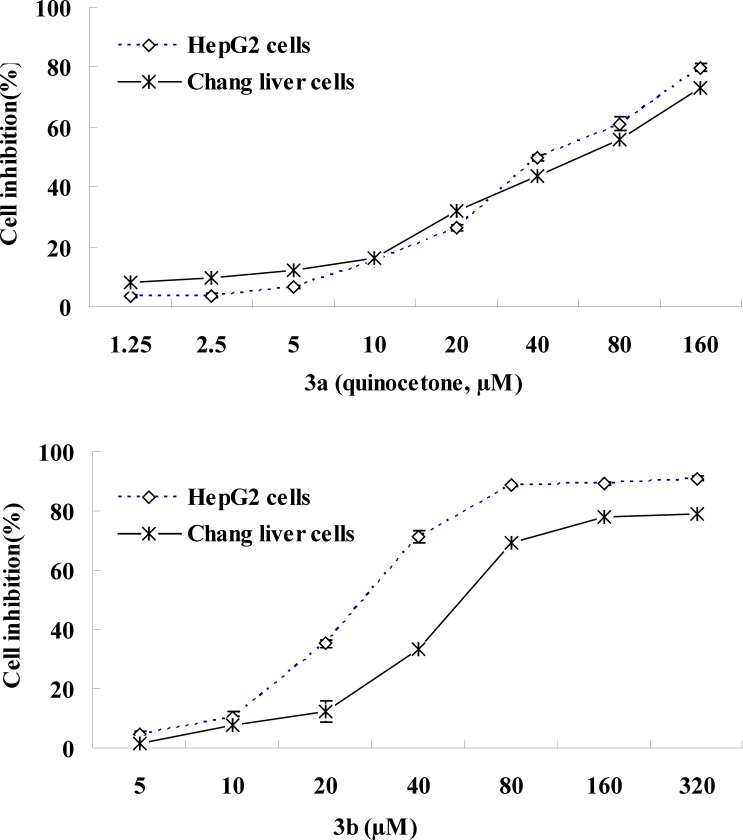
Cytotoxicity of 3a (quinocetone) and 3b in HepG2 cells and Chang cells. Cells were incubated with the drugs at indicated concentrations for 24 h. Cell proliferation was measured by MTT assay. Control value was taken as 100%.

**Table 1:** Results of antibacterial activities of quinoxaline-1,4-dioxides and their deoxygenations

**compounds**	**MIC values** **（** **μg/mL** **）**				
	***Escherichia coli *** ** ATCC25922**	***Salmonella pullorum***	***Aeromonas hydrophila***	***Clostridium perfringen***	***Staphyloccocus aureus*** ** ATCC25923**
olaquindox	21.04	84.16	21.04	67.328	42.08
2	4.36	17.44	17.44	8.72	17.44
4	>500	476.16	119.04	>500	>500
6	94.72	189.44	94.72	94.72	94.72
7	>500	>500	337.92	>500	>500
8	4.96	19.84	39.68	4.96	39.68
9	>500	>500	138.24	>500	>500
10	>500	>500	481.28	>500	>500
11	56.32	450.56	225.28	225.28	450.56
3a	97.92	48.96	48.96	195.84	24.48
5a	>500	>500	>500	>500	>500
3b	103.04	25.76	206.08	51.52	12.88
5b	>500	>500	371.2	>500	>500
3c	103.04	103.04	103.04	25.76	25.76
5c	>500	>500	371.2	>500	>500
3d	>500	56.16	449.28	>500	449.28
5d	>500	>500	>500	>500	>500
3e	449.28	224.64	112.32	449.28	449.28
5e	>500	>500	>500	>500	>500
3f	>500	>500	>500	26.88	13.44
5f	>500	>500	>500	>500	>500
3g	>500	>500	>500	435.2	27.2
5g	>500	>500	>500	>500	>500
3h	446.72	>500	223.36	446.72	446.72
5h	>500	>500	>500	>500	>500

**Table 2:** A comparison of the cytotoxic effects of quinoxaline-1,4-dioxide derivatives and their deoxygenation on HepG2 and Chang liver cells

**Compound**	**IC**_50_ **values (µM)** a **24h HepG2**	**IC**_50_ ** values (µM)** a **24h Chang liver cells**
olaquindox	892.78±14.66	815.21±31.24
2	287.64±9.74	262.58±3.11
4	571.88±36.20	671.59±23.24
6	240.27±34.81	299.44±6.72
7	440.89±12.31	468.56±5.26
8	217.40±3.48	217.26±3.52
9	547.01±7.71	609.94±18.37
10	2487.44±133.18	2117.55±90.13
11	465.41±28.81	452.04±6.86
3a	49.31±1.94	63.30±5.55
5a	451.23±11.06	532.24±7.66
3b	30.46±1.30	67.99±2.90
5b	525.53±20.92	463.02±9.91
3c	102.44±4.13	191.98±10.30
5c	273.98±11.64	361.36±10.23
3d	380.14±10.58	410.60±17.09
5d	461.04±4.49	499.30±8.75
3e	449.32±19.40	535.99±22.95
5e	605.91±34.56	590.09±2.22
3f	99.68±2.97	86.91±6.30
5f	653.34±31.97	531.31±19.28
3g	102.54±4.08	113.73±4.59
5g	532.68±24.77	688.78±46.18
3h	394.94±14.59	508.74±14.78
5h	626.29±25.35	567.27±26.67

a The IC_50_ figure is the concentration of the compound required to reduce the number of viable cells by 50%. The highest concentration used is maximum values which can be dissolved in DMEM.

**Table 3:** The protonated molecular ions of the compounds metabolites

**Compound**	**parent drug** **[M+H]** ^+^ ** (** ***m/z*** **)**	**Metabolites** **[M+H]** ^+^ **(*m/z*)**
3a	307	309(Q1), 311(Q2), 295(Q3), 293(Q4), 277(Q5)
5a	275	291(DQ1), 293 (DQ2), 277(DQ3) , 279 (DQ4), 295(DQ5)
6	297	299(QA1), 283(QA2), 267(QA3), 301(QA4), 285(QA5), 265(QA6)
8	249	233(MQ1), 233(MQ2), 217(MQ3)
olaquindox	264	248(O1)

The MS instrument was used for analysis operating in a positive ion mode. The main parameters were optimized by infusing 1μg/mL of various pharmaceuticals standard dissolved in acetonitrile at 10 μL/min using a needle (Hamilton, Switzerland). Nitrogen was used for desolvation gas (400 L/h) and nebulizing gas (50 L/h). The source temperature was held at 100˚C. The capillary and cone voltages were 3.5 Kv and 30 v, respectively. Argon was used for a collision gas at a pressure of 3.3×10^-3 ^mbar. The spectra was recorded in the range *m/z* 50~500 for full scan MS analysis. 

 Based on comparing their retention times, full scan, product ion scan to available authentic standards, the metabolites of test compounds were identified. Multiple reaction monitoring (MRM) was taken as a supplement to confirm the conclusion. Selected ion recording (SIR) extracted chromatogram of possible metabolites also was used. Setting molecular weight of the SIR is based on the chemical structure of each compound and literature. System control and data acquisition were completed by using the software Masslynx 4.0 (Waters, Milford, MA, USA).

## Results

*Synthesis of test compounds*

The chemistry work was undertaken in the Key Laboratory of Veterinary Drug Safety Evaluation and Residues Research. The synthesis of the desired compounds which include quinocetone 3a, quinocetone structure similar compounds 3b–h and 6, their deoxygenation 5a–h, as well as 2, 4, 7, 8, 9, 10 and 11 are presented in Figure 2. 

*2-acetyl-3-methylquinoxaline-1,4-dioxide 2*. 

Yield: (60 %); [M+H]^+^ m/z : 219.3, C_11_H_10_N_2_O_3_; mp: 151.2～153.5 ℃; IR (KBr) νmax/cm^-1^: 1700.2, 1515.2, 1329.1, 1274.5, 1096.8, 1050.3, 829, 775.5, 614.6; ^1^HNMR (400 MHz, DMSO-*d6*), (δ: ppm): 8.47~8.39 (m, 2H), 7.99~7.91 (m,2H), 2.61(s,3H), 2.33(s,3H)。^13^C NMR (400 MHz, CDCl3) (δ: ppm): 194.2, 139.7, 138.9, 137.8, 136.8, 132.5, 131.5, 120.2, 119.9, 29.9, 13.8. 

*2-(3-aryl-2-propenyl)-3-methylquinoxaline-1,4-dioxides 3a–h*.

Data for 3a (quinocetone). Yield: (49 %); [M+H]^+^ m/z: 307.1, _18_H_14_N_2_O_3_; mp: 183.2～185.3 ℃; IR (KBr) νmax/cm^-1^: 3098.1, 1660.8, 1623.4, 1331.8, 1095.7, 1044.7, 768.8, 636.1; ^1^HNMR (400 MHz, DMSO-*d6*), (δ: ppm): 8.52(d, j = 8.0 Hz, 1H), 8.41(d, j = 8.0 Hz, 1H), 8.02~7.90(m, 2H), 7.83(d, j = 44 .0Hz 1H), 7.72(d, j = 8.0 Hz, 2H), 7.42~7.50 (m,3H), 7.22(d, j = 16.0 Hz 1H), 2.33(s,3H). ^13^C NMR (400 MHz, CDCl3) (δ: ppm):185.8, 147.3, 139.9, 139.0, 137.9, 136.9, 133.6, 132.5, 131.7, 131.4, 129.0, 128.9, 124.7, 120.2, 14.2. 

Data for 3b. Yield: (36 %); [M+H]^+^ m/z: 323.3, C_18_H_14_N_2_O_4_; mp: 188.7～192 ℃; IR (KBr) νmax/cm^-1^: 3421.8, 3089.1, 1644.6, 1612, 1346.7, 1328.1, 1215.4, 1044, 788.3; ^1^HNMR (400 MHz, DMSO-*d6*), (δ: ppm): 9.65(s,1H), 8.52(d, j = 8.0 Hz,1H), 8.41(d, j = 8.0 Hz,1H), 7.96~7.92(m,2H), 7.75(d, j = 16.0 Hz,1H), 7.36~7.05(m,4H), 6.86(d, j = 8.0 Hz,1H), 2.33(s,3H). ^13^C NMR (400 MHz, CDCl3) (δ: ppm): 187.6, 158.2, 149.5, 139.3, 138.9, 138.0, 137.1, 135.7, 132.7, 131.8, 130.4, 125.7, 120.5, 120.0, 119.2, 115.7, 14.4. 

Data for 3c. Yield: (52 %); [M+H]^+^ m/z: 323.1, C_18_H_14_N_2_O_4_; mp: 214.8～219.5 ℃; IR (KBr) νmax/cm^-1^: 3421.8, 3085.8, 1596.7, 1570.4, 1326.4, 1090.5, 791.8, 750.5; ^1^HNMR (400 MHz, DMSO-*d6*), (δ: ppm): 10.26(s, 1H), 8.50(d, j = 8.0 Hz,1H), 8.41(d, j = 8.0 Hz,1H), 7.96~7.92(m,2H), 7.64(d, j = 16.0 Hz, 1H), 7.58(d, j = 8.0 Hz, 2H), 6.98(d, j = 8.0 Hz，1H), 6.7,8 (d, j = 16.0Hz，2H), 2.32(s, 3H). ^13^C NMR (400 MHz, CDCl3) (δ: ppm):187.6, 158.3, 144.9, 139.3, 139.2, 137.9, 137.0, 133.4, 132.8, 131.8, 131.2, 125.7, 120.9, 120.0, 119.9, 116.9, 14.4. 

Data for 3d. Yield: (42 %); [M+H]^+^ m/z: 352.4, C_18_H_13_N_3_O_5_; mp: 201.8～203.9 ℃; IR (KBr) νmax/cm^-1^: 3074.2, 1682.9, 1608.5, 1531.5, 1332.8, 1096.4, 823.8, 772.9; ^1^HNMR (400 MHz, DMSO-*d6*), (δ: ppm): 8.54(d, j = 8.0 Hz，1H), 8.48(s,1H), 8.42(d, j = 8.0 Hz，1H), 8.25(d, j = 4.0 Hz，2H), 8.05~7.93(m,3H), 7.75~7.70(m,1H), 7.43(d, j = 16.0 Hz，1H), 2.27(s,3H). ^13^C NMR (400 MHz, CDCl3) (δ: ppm): 187.8, 148.7, 146.5, 139.3, 138.1, 137.1, 136.1, 134.6, 132.9, 131.9, 130.9, 128.2, 125.9, 124.6, 120.1, 14.4. 

Data for 3e. Yield: (56 %); [M+H]^+^ m/z: 352.1, C_18_H_13_N_3_O_5_; mp: 213～217.9 ℃; IR (KBr) νmax/cm^-1^: 3081.1, 1682.4, 1613.6, 1521.2, 1342.2, 1098.4, 754.5; ^1^HNMR (400 MHz, DMSO-*d6*), (δ: ppm): 8.52(d, j = 8.0 Hz，1H), 8.41(d, j = 8.8 Hz，1H), 8.24(d, j = 9.2 Hz，2H), 7.99~7.90(m,5H), 7.40(d, j = 16.8 Hz，1H), 2.34(s,3H). 

Data for 3f. Yield: (41 %); [M+H]+ m/z: 337.3, C19H16N2O4; mp: 163.6～166.8 ℃; IR (KBr) νmax/cm^-1^: 2867.1, 1592.6, 1336.5, 1238.5, 1180.8, 1028.1, 829.7, 782.8; ^1^HNMR (400 MHz, DMSO-*d6*), (δ: ppm): 8.51(d, j = 8.0 Hz，1H), 8.41(d, j = 8.8 Hz，1H), 7.98~7.93(m,2H), 7.78~7.68 (m,3H), 7.06(d, j = 16.0 Hz，1H), 6.97(d, j = 8.8 Hz，2H), 3.77(s,3H), 2.32(s,3H). 

Data for 3g. Yield: (47 %); [M+H]^+^ m/z: 341.5, C_18_H_13_ClN_2_O_3_; mp: 182.8～184.5 ℃; IR (KBr) νmax/cm^-1^: 3079.8, 1678.4, 1607, 1335.9, 1098.8, 1011.8, 813.5, 760.3; ^1^HNMR (400 MHz, DMSO-*d6*), (δ: ppm): 8.51(d, j = 8.4 Hz，1H), 8.40(d, j = 8.0 Hz，1H), 7.98~7.93(m,2H), 7.83~7.78 (m,3H), 7.48(d, j = 8.4 Hz，2H), 7.23(d, j = 16.4 Hz，1H), 2.33(s,3H). ^13^C NMR (400 MHz, CDCl3) (δ: ppm):185.5, 145.4, 139.9, 137.9, 137.7, 136.9, 132.6, 132.1, 131.5, 130.0, 129.3, 128.9, 124.9, 120.2, 115.9, 14.1. 

Data for 3h. Yield: (40 %); [M+H]^+^ m/z: 350.3, C_20_H_19_N_3_O_3_; mp: 217.8～219.5 ℃; IR (KBr) νmax/cm^-1^: 2834, 1629.3, 1577.1, 1337.1, 1237.7, 1183.2, 1044.1, 820, 774.4; ^1^HNMR (400 MHz, DMSO-*d6*), (δ: ppm): 8.51(d, j = 12.0 Hz，1H), 8.41(d, j = 8.0 Hz，1H), 8.03~7.91(m,2H), 7.62(d, j = 16.0 Hz，1H), 7.54(d, j = 8.0 Hz，2H), 6.87(d, j = 16.0 Hz，1H), 6.68(d, j = 8.0 Hz，2H), 2.97(s,6H), 2.32(s,3H). ^13^C NMR (400 MHz, CDCl3) (δ: ppm): 185.2, 152.8, 149.2, 139.9, 139.8, 137.7, 137.1, 132.1, 131.4, 131.2, 121.2, 120.4, 120.1, 119.4, 117.7, 40.2, 14.3. 

*2-acetyl-3-methylquinoxaline 4.*

Yield: (82 %); [M+H]^+^ m/z: 187.1, C_11_H_10_N_2_O; mp：90.3～91.6 ℃; IR (KBr) νmax/cm^-1^: 2910.5, 1696.9, 1562.2, 1363, 1190.8, 1123.4, 1056.9, 937.9, 776.2, 650; ^1^HNMR (400 MHz, DMSO-*d6*), (δ: ppm): 8.12(d, j = 8.0 Hz,1H), 8.11(d, j = 8.0 Hz,1H), 8.04~8.01 (m,1H), 7.937.84(m,1H), 2.81(s,3H), 2.72(s,3H).^ 13^C NMR (400 MHz, CDCl3) (δ: ppm): 201.1, 152.9, 147.0, 142.5, 139.7, 131.9, 129.7, 129.5, 128.3, 27.7, 24.3. 

*2-(3-aryl-2-propenyl)-3-methylquinoxaline 5a–h*.

Data for 5a (deoxyquinocetone)**. **Yield: (52 %); [M+H]^+^ m/z: 275.1, C_18_H_14_N_2_O; mp :153.6～156.8 ℃; IR (KBr) νmax/cm^-1^: 2998.1, 1674.1, 1618.1, 1592, 1448.6, 1327.9, 1314.1, 979.1, 775.7, 732.1; ^1^HNMR (400 MHz, DMSO-*d6*), (δ: ppm): 8.18(d, j = 8.4 Hz,1H), 8.07(d, j = 8.0 Hz,1H), 7.87~7.84(m,3H), 7.88~7.74(m,3H), 7.47~7.45(s,3H), 2.82(s,3H). ^13^C NMR (400 MHz, CDCl3) (δ: ppm): 201.6, 191.0, 153.5, 153.4, 148.6, 146.8, 145.6, 131.7, 130.8, 129.7, 129.6, 128.9, 128.8, 128.4, 128.2, 127.8, 123.3, 24.0. 

Data for 5b**. **Yield: (41 %); [M+H]^+^ m/z: 291.2, C_18_H_14_N_2_O_2_; mp : 137.8～140.3 ℃; IR (KBr) νmax/cm^-1^: 3154.3, 1670, 1597.4, 1581.1, 1475.5, 1323.7, 981.9, 785.2, 748.4; ^1^HNMR (400 MHz, DMSO-*d6*), (δ: ppm): 9.68(s,1H), 8.00(d, j = 4.4 Hz,1H), 7.91(d, j = 8.0 Hz,1H), 7.88~7.84(m,2H), 7.78(d, j = 16.0 Hz,1H), 7.67(d, j = 16.0 Hz,1H), 7.23~7.20(m,2H), 7.14(s,1H), 6.86(d, j = 8.0 Hz, 1H), 2.27(s,3H).^ 13^C NMR (400 MHz, CDCl3) (δ: ppm): 190.7, 182.9, 155.9, 148.5, 145.0, 143.4, 142.2, 139.8, 136.5, 131.9, 130.1, 129.8, 129.7, 128.3, 123.7, 121.8, 117.9, 114.9, 23.9. 

Data for 5c**. **Yield: (45 %); [M+H]^+^ m/z: 291.0, C_18_H_14_N_2_O_2_; mp : 182.6～184.3 ℃; IR (KBr) νmax/cm^-1^: 2926.2, 1697.1, 1539.1, 1516, 1498.7, 1280.9, 1171.2, 795.6, 759.9; ^1^HNMR (400 MHz, DMSO-*d6*), (δ: ppm): 9.81(brs,1H), 8.16~8.14(m,1H), 8.04~8.00(m,1H), 7.86~7.76(m,2H), 7.67~7.51(m,2H), 7.22(d, j = 8.0 Hz,1H), 7.20~7.18 (m,1H), 6.98~6.94(m,2H), 2.60(s,3H).^ 13^C NMR (400 MHz, CDCl3) (δ: ppm): 201.3, 155.8, 153.4, 153.0, 149.2, 142.6, 141.2, 139.8, 132.0, 131.9, 131.6, 129.8, 129.7, 129.6, 129.5, 128.4, 124.0, 120.9, 116.5, 24.3. 

Data for 5d**. **Yield: (40 %); [M+H]^+^ m/z: 320.1, C_18_H_13_N_3_O_3_; mp : 169～172.8 ℃; IR (KBr) νmax/cm^-1^: 3053.2, 1675, 1609.4, 1524.7, 1353.4, 984.2, 768.6, 727.8; ^1^HNMR (400 MHz, DMSO-*d6*), (δ: ppm): 8.60(s,1H), 8.31~8.18(m,3H), 8.08~7.89(m,5H), 7.75~7.73 (m,1H), 2.83(s,3H). 

Data for 5e**. **Yield: (53 %); [M+H]^+^ m/z: 320.3, C_18_H_13_N_3_O_3_; mp : 204.6～208.2 ℃; IR (KBr) νmax/cm^-1^: 3019.1, 1672.9, 1607.7, 1511, 1344.1, 1120, 975.2, 847.7, 776.4, 761.5; ^1^HNMR (400 MHz, DMSO-*d6*), (δ: ppm): 8.27(d, j = 8.4 Hz,2H), 8.19(d, j = 8.0 Hz,1H), 8.11~8.09(m,4H), 7.96~7.93(m,1H), 7.90~7.86(m,2H), 2.85(s,3H). ^13^C NMR (400 MHz, CDCl3) (δ: ppm): 189.9, 153.8, 147.4, 142.6, 141.7, 139.6, 136.6, 134.4, 132.2, 129.9, 129.8, 128.5, 125.6, 124.7, 122.8, 24.2. 

Data for 5f**. **Yield: (52 %); [M+H]^+^ m/z: 305.1, C_19_H_16_N_2_O_2_; mp : 143.8～145.5 ℃; IR (KBr) νmax/cm^-1^: 2942.2, 1664, 1592.1, 1512.1, 1260.4, 1178.5, 988, 820.5, 749.9; ^1^HNMR (400 MHz, DMSO-*d6*), (δ: ppm): 8.16(d, j = 8.0 Hz,1H), 8.05(d, j = 8.0 Hz,1H), 7.93~7.89(m,2H), 7.85~7.63(m,4H),7.00(d, j = 8.8 Hz,2H), 3.79(s,3H), 2.79(s,3H). 

Data for 5g**. **Yield: (45 %); [M+H]^+^ m/z: 309.1, C_18_H_13_ClN_2_O; mp : 174.5～176.1 ℃; IR (KBr) νmax/cm^-1^:3108.6, 1740.3, 1522.6, 1352, 1332.7, 1244.8, 1061.2, 1014.1, 777.9, 633.9; ^1^HNMR (400 MHz, DMSO-*d6*), (δ: ppm): 8.18(d, j = 8.0 Hz,1H), 8.07(d, j = 8.0 Hz,1H), 7.93~7.73(m,6H), 7.51(d, j = 8.4 Hz,2H), 2.82(s,3H). 

Data for 5h**. **Yield: (43 %); [M+H]^+^ m/z: 318.2, C_20_H_19_N_3_O; mp : 160～162.1 ℃; IR (KBr) νmax/cm^-1^: 2931.4, 1653.5, 1581.3, 1373.4, 1189.8, 1027.1, 1000.3, 808.9, 750; ^1^HNMR (400 MHz, DMSO-*d6*), (δ: ppm): 8.14(d, j = 8.0 Hz,1H), 8.05(d, j = 8.0 Hz,1H), 7.89~7.83(m,2H), 7.63~7.59(m,3H), 7.43(d, j = 12.0 Hz,1H), 6.71(d, j = 8.4 Hz,2H), 2.98(s,6H), 2.76(s,3H).^ 13^C NMR (400 MHz, CDCl3) (δ: ppm): 191.5, 153.2, 152.2, 150.2, 147.4, 142.2, 139.7, 131.1,130.9, 129.6, 129.4, 128.4,122.4,118.5,111.7, 40.0, 23.7.

*3-methyl-2-(3-fura-2-propenoyl)-quinoxaline-1,4-dioxide 6*. 

Yield: (62 %); [M+H]^+^ m/z: 297.2, C_16_H_12_N_2_O_4_; mp : 177～178.5 ℃; IR (KBr) νmax/cm^-1^: 3109.3, 1670.2, 1609.1, 1551.1, 1332.6, 1274.9, 1099.5, 1027.9, 995.9, 764.3; ^1^HNMR (400 MHz, DMSO-*d6*), (δ: ppm): 8.48(d, j = 8.8 Hz,1H), 8.40(d, j = 8.4 Hz,1H), 7.99~7.90(m,3H), 7.68(d, j = 16.0 Hz,1H), 6.97(d, j = 3.2 Hz,1H), 6.79(d, j = 16.0 Hz,1H), 6.67(d, j = 2.0 Hz,1H), 2.32(s,3H).^ 13^C NMR (400 MHz, CDCl3) (δ: ppm): 185.2, 150.5, 146.3, 139.8, 139.1, 137.8, 136.9, 132.6, 132.4, 131.3, 121.9, 120.2, 120.1, 118.4, 113.1, 14.1.

*3-methyl-2-(3-fura-2-propenoyl)-quinoxaline 7*. 

Yield: (45 %); [M+H]^+^ m/z: 265.4, C_16_H_12_N_2_O_2_; mp : 133.6～134.8 ℃; IR (KBr) νmax/cm^-1^: 3041.1, 1670.9, 1606.3, 1552, 1475.1, 1315.6, 1200.5, 970.6, 757, 697; ^1^HNMR (400 MHz, DMSO-*d6*), (δ: ppm): 8.18(d, j = 8.4 Hz,1H), 8.06(d, j = 8.0 Hz,1H), 7.94~7.86 (m,3H), 7.64~7.56(m,2H), 7.09(d, j = 3.2 Hz,1H), 6.69(s,1H), 2.83(s,3H).^ 13^C NMR (400 MHz, CDCl3) (δ: ppm): 190.7, 153.5, 151.7, 148.5, 145.4, 142.4, 139.7, 131.7, 131.2, 129.8, 129.5, 128.4, 120.9, 116.8, 112.8, 24.0.

*3-methyl-2-quinoxaline-2-carboxylate-1,4-dioxide 8*.

 Yield: (60 %); [M+H]^+^ m/z : 249.3, C_12_H_12_N_2_O_4_; mp : 136.0～137.1 ℃; IR (KBr) νmax/cm^-1^: 3040.1, 1669.7, 1603.9, 1565.1, 1487.9, 1404.1, 1327.9, 1092.9, 980.2, 820.8, 765.1; ^1^HNMR (400 MHz, DMSO-*d6*), (δ: ppm): 8.44~8.37 (m,2H), 7.98~7.91(m,2H), 4.47(q, j = 8.0 Hz, 2H), 2.40(s,3H), 1.33(t, j = 8.0 Hz,3H).^ 13^C NMR (400 MHz, CDCl3) (δ: ppm): 159.8, 138.9, 137.8, 136.8, 135.5, 132.5, 131.4, 120.3, 120.0, 63.6, 14.3, 13.9.

*3-methyl-2-quinoxaline-2-carboxylate 9*. 

Yield: (66 %); [M+H]^+^ m/z : 217.3, C_12_H_12_N_2_O_2_; mp : 78.6～80.1 ℃; IR (KBr) νmax/cm-1:2971.9, 1717.1, 1481.7, 1368.7, 1316.2, 1271.1, 1123.5, 1082.6, 851.0, 769.4; ^1^HNMR (400 MHz, DMSO-*d6*), (δ: ppm): 8.12(d, j = 8.0 Hz，1H), 8.04(d, j = 8.0 Hz，1H), 7.93~7.89 (m,1H), 7.86~7.79(m,1H), 4.42(q, j = 8.0 Hz, 2H), 2.80(s,3H), 1.35(t, j = 8.0 Hz,3H). ^13^C NMR (400 MHz, CDCl3) (δ: ppm): 165.6, 152.7, 144.4, 142.4, 139.8, 131.7, 129.7, 128.4, 62.4, 23.6, 14.2.

*3-methyl-quinoxaline-2-carboxylic acid 10*. 

Yield: (32 %); [M+H]^+^ m/z : 189.1, C_10_H_8_N_2_O_2_; mp : 315.6～320 ℃; IR (KBr) νmax/cm^-1^: 2788.5, 2502.5, 1716.8, 1567.8, 1488, 1326, 1232.9, 837, 77,3.8, 765.4, 748.7, 709.7; ^1^HNMR (400 MHz, DMSO-*d6*), (δ: ppm): 8.09~7.89 (m,2H), 7.87~7.79(m,2H), 2.79(s,3H). ^13^C NMR (400 MHz, CDCl3) (δ: ppm): 163.3, 154.7, 143.9, 139.2, 138.4, 132.9, 130.5, 128.9, 128.6, 24.3.

*3-methyl-quinoxaline-1,4-dioxide 11.*

Yield: (44.6 %); [M+H]^+^ m/z : 177.1, C_9_H_8_N_2_O_2_; mp: 173.5~176.8 ℃; IR (KBr) νmax/cm-1:3028.5, 1540.9, 1505.9, 1352.3, 1335.5 1237.9, 1097.3, 963.4, 777.0, 741.3, 640.1; ^1^HNMR (400 MHz, DMSO-*d6*), (δ: ppm): 8.75(s,1H), 8.40~8.37(m,2H), 7.93~7.85(m,2H), 2.42(s,3H).^ 13^C NMR (400 MHz, CDCl3) (δ: ppm): 141.2, 138.0, 137.3, 131.9, 131.1, 130.8, 120.1, 119.9, 15.7.

*In-vitro antibacterial activity*

In this study, activity of quinoxaline-1, 4-dioxides and their deoxygenations was tested against selected Gram-negative bacteria (*Escherichia coli, Salmonella pullorum *and *Aeromonas hydrophila*), Gram-positive bacteria (*Staphyloccocus aureus* and* Clostridium perfringen*). The *in-vitro *antibacterial activity was based on the MIC results: Strong antibacterial activity, if MIC is less than 50 μg/mL; Moderate, if MIC is in between 50 and 250 μg/mL; No activity, if MIC is more than 500 μg/mL. The obtained results are presented in Table 1.

The results of antibacterial testing revealed that compounds 2, 6, 8, 3a, 3b, 3c and olaquindox to have strong or moderate activity against both Gram-positive and Gram-negative bacteria. Compounds 4, 9, 11, 3d, 3e, 3f, 3g and 3 h possessed little or no activity against Gram-positive and Gram-negative bacteria. Compounds did not show significant differences against Gram-positive and Gram-negative bacteria. The other tested compounds showed no activity. In addition, olaquindox can kill *Aeromonas hydrophila* at 84.16 μg/mL, MBC values of other compounds were greater than 500 μg/mL, and all compounds have no killing activity for other bacteria.

*Toxicity against the human liver cells*

In order to investigate the effects of chemical structure on cytotoxicity for quinoxaline-1,4-dioxide derivatives and their deoxygenation, all of the compounds were evaluated for their inhibitory activity against human liver cells (HepG2 and Chang liver cells) proliferation using the MTT assay. The IC_50_ values obtained for the compounds after 24 h of incubation are shown in Table 2.

The IC_50_ values of the following compounds are less than 100 µM (significant cytostatic properties) namely 3a (49), 3b (30), 3c (102), 3f (99) and 3g (102) in the HepG2 assay and 3a (63), 3b (68) and 3f (87) in the Chang liver cells screen. The IC_50_ figures are more than 300 µM for the remaining compounds in both tests with the exception of 2, 6, 8. 

*Metabolic pathways in HepG2 cells*

According to comparison of both total ion chromatogram (TICs) and selected ion reaction spectra (SIRs) of pretreated samples (Figure 3), taking spectra at the top of the peaks in the TICs and detecting low levels of possible metabolites according to SIR spectras got the protonated molecular ions of quinocetone 3a metabolites. The protonated molecular ions of these metabolites were *m/z* 309(Q1), *m/z* 311(Q2), *m/z* 295(Q3),* m/z* 293(Q4) and* m/z *277(Q5), respectively. 

To further elucidate the structure of these metabolites, the accurate MS^2^ spectra of the metabolites was simultaneously measured in one experiment. The fragment ions employed in the identification of metabolites are summarized (data not shown). The analysis results indicated that the metabolites could be divided into three categories: reduction, hydroxylation, and a combination of reduction and hydroxylation. Therefore, we concluded that Q1 was product of alkenyl reduction and hydroxylation, Q2 was product of alkenyl reduction, Q3, Q4, Q5 were the N→O group reduction metabolites of quinocetone. Combining above results with common metabolic pathways, we tentatively identified the five main metabolites as Q1, Q2, Q3, Q4 and Q5 for quinocetone 3a (Figure 4). With the same method, we obtained the information of metabolites of deoxyquinocetone 5a, olaquindox, 3-methyl-2-(3-fura-2-propenoyl)-quinoxaline -1,4-dioxide 6, 3-methyl-2-quinoxaline-2- carboxylate-1,4-dioxide 8. The protonated molecular ions of the compounds metabolites were showed in Table 3 respectively.

## Discussion

The synthesis of the desired compounds which include quinocetone 3a, quinocetone structure similar compounds 3b–h and 6, their deoxygenation 5a–h, as well as 2, 4, 7, 8, 9, 10 and 11 are presented in Figure 2. Benzofurazan-1-oxide 1, which was prepared by hypochlorite oxidation of 2-nitroaniline according to a literature procedure (18, 19), was reacted with acetylacetone to yield 2-acetyl-3-methylquinoxaline-1,4-dioxide 2 (20). Deoxygenation of quinoxaline N-oxides are prepared by the method modified from Haddadin›s methods with reduction of sodium dithionite (21). A number of aryl aldehydes were condensed with 2 or 4 leading to 3a–h, 5a–h, 6 and 7. 1H NMR spectroscopy revealed that the olefinic double bond in the desired compounds possesses the E-configuration. The observed molecular weight that losses for all deoxygenation is in good agreement with calculated values which showed the compounds were successfully synthesised.

Derivatives of series quinocetone 3a, quinocetone structure similar compounds 3b–h and their deoxygenation 5a–h, possessed a 3-aryl-2-propenyl group in the 2-position of the quinoxaline ring which were unsubstituted or substituted by different electronic substituents in the R1, R2, R3-position of arylidene aryl ring. Chloro and nitro were used as electron-withdrawing groups, whereas dimethylamino, hydroxy and methoxy were used as electron donating groups. Compounds 2, 3a-h, 6, 8 and olaquindox all have the same 2-acetyl-3-methylquinoxaline-1, 4-dioxide group structure, and 4, 5a-h, 7, 9, and 10 were the deoxygenation of 2-acetyl-3-methylquinoxaline-1,4-dioxide group structure, while olaquindox, 2, 8 and 11 were bereft molecules of an alkylating function from series 3. 3-aryl-2-propenyl group was unique structural features for quinocetone 3a, its structure similar compounds 3b–h and their deoxygenation 5a–h.

The antibacterial activity of certain quinoxaline 1, 4-dioxides has been described since 1940s (22). Previous studies indicated that antibacterial activity of quinoxaline-1,4-dioxide derivatives are dependent upon the presence of their N-oxide groups (12). It completely agrees with our findings, Because due to deoxygenation, completely reduced derivatives of quinoxaline-1, 4-dioxide derivatives lose their antibacterial activity. Although 3d-h possessed N-oxide groups and 3-aryl-2-propenyl group, their antibacterial activity was not as high as quinocetone 3a. The results suggested different electronic substituents in the R1, R2, R3-position of arylidene aryl ring that could influence the biology activity just the same as N-oxide groups reduction. 

Accumulating evidence has suggested that the toxicity of quinoxaline-1, 4-dioxide derivatives is dependent on the presence of their N-oxide groups because the reduction reaction of N-oxide groups can directly generate ROS (23, 24). Whereas, Das (19) synthesized a series of 2-(3-Aryl-2-propenoyl)-3- methylquinoxaline-1,4-dioxides and investigated their antineoplastic effect and revealed that the majority of the compounds displayed a greater toxicity to neoplastic than normal cells, and the different electronic substituents in the R1, R2, R3-position of arylidene aryl ring should influence the cytostatic potency. Our results showed that cancers HepG2 cells were slightly more sensitive to quinoxaline derivatives than normal human Chang liver cells, and proved that quinocetone 3a have grate cytotoxicity again, and displayed that the attachment of ortho-hydroxy moiety to the heterocycle (3b) had more toxicity potency (Figure 5). Meanwhile the date revealed that derivatives of series 3a-h, possessed a 3-aryl-2-propenyl group in the 2-position of the quinoxaline ring, have more serious cytotoxicity in general terms than the attachment of other moiety, such as 2, 8 and olaquindox, and supported the hypothesis that 2-propenoyl is the main toxiccophore after N-oxide groups in the chemical structure of quinocetone.

Drug metabolizing is of paramount importance in drug detoxification (25, 26). To elucidate metabolic characteristics of quinoxaline-1,4-dioxide derivatives *in-vitro* test systems, metabolites of olaquindox, quinocetone 3a, 3-methyl-2-(3-fura-2-propenoyl) -quinoxaline-1,4-dioxide 6, 2-(3-phenyl-2-propenyl) -3-methylquinoxaline 5a and 3-methyl-2-quinoxaline -2-carboxylate- 1,4-dioxide 8 were identified by high-performance liquid chromatography using electrospray ionization tandem mass spectrometry. The major metabolic pathways of the compound’s metabolism in HepG2 cells revealed that in addition to N-oxide groups, 2-propenoyl was another major metabolic site. Reduction 2-propenoyl in HepG2 cells suggested the moiety is one of major toxiccophore.

## Conclusion

In this study, quinocetone and other new quinoxaline-1,4-dioxide derivatives including quinocetone structure similar compounds 3b-h and their deoxygenation were synthesized by the Beirut reaction and reduction of sodium dithionite. The results of antibacterial activity analyses demonstrated that N-oxide groups were the major antibacterial activity and the activity was also influenced by electronic substituents on arylidene aryl ring. Quinocetone and its hydroxylated compound 3b have significant cytotoxicity and N→O groups reduction was the major metabolic pathway showed N-oxide groups were the major activity groups in the chemical structure. Meanwhile, comparing possessed 2-propenyl group and other quinoxaline-1,4-dioxide derivatives, 2-propenyl group contributed to the cytostatic properties and was a major metabolic site in cell revealed 2-propenoyl is another major toxiccophore.

## Conflict of Interest

The authors declare that they have no competing interests.
